# Maturational delay and asymmetric information flow of brain connectivity in SHR model of ADHD revealed by topological analysis of metabolic networks

**DOI:** 10.1038/s41598-020-59921-4

**Published:** 2020-02-21

**Authors:** Seunggyun Ha, Hyekyoung Lee, Yoori Choi, Hyejin Kang, Se Jin Jeon, Jong Hoon Ryu, Hee Jin Kim, Jae Hoon Cheong, Seonhee Lim, Bung-Nyun Kim, Dong Soo Lee

**Affiliations:** 1 Department of Nuclear Medicine, Seoul National University College of Medicine, Seoul, Republic of Korea; 2 Division of Nuclear Medicine, Department of Radiology, Seoul St. Mary’s Hospital, College of Medicine, The Catholic University of Korea, Seoul, Republic of Korea; 3 Biomedical Research Institute, Seoul National University Hospital, Seoul, Republic of Korea; 4 BK21 Plus Global Translational Research on Molecular Medicine and Biopharmaceutical Sciences, Seoul National University, Seoul, Republic of Korea; 5 Department of Oriental Pharmaceutical Science, College of Pharmacy, Kyung Hee University, Seoul, Republic of Korea; 6 Department of Life and Nanopharmaceutical Science, College of Pharmacy, Kyung Hee University, Seoul, Republic of Korea; 7 Department of Pharmacy, Uimyung Research Institute for Neuroscience, Sahmyook University, Seoul, Republic of Korea; 8 Department of Mathematical Sciences, Seoul National University, Seoul, Republic of Korea; 9 Division of Child and Adolescent Psychiatry, Department of Psychiatry, Seoul National University College of Medicine, Seoul, Republic of Korea; 10 Department of Molecular Medicine and Biopharmaceutical Sciences, Graduate School of Convergence Science and Technology, and College of Medicine or College of Pharmacy, Seoul National University, Seoul, Republic of Korea

**Keywords:** Network models, Network models, ADHD

## Abstract

Attention-deficit hyperactivity disorder (ADHD) is a complex brain development disorder characterized by hyperactivity/impulsivity and inattention. A major hypothesis of ADHD is a lag of maturation, which is supported mainly by anatomical studies evaluating cortical thickness. Here, we analyzed changes of topological characteristics of whole-brain metabolic connectivity in twelve SHR rats selected as ADHD-model rats by confirming behavior abnormalities using the marble burying test, open field test, and delay discounting task and 12 Wistar Kyoto rats as the control group, across development from 4 weeks old (childhood) and 6 weeks old (entry of puberty). A topological approach based on graph filtrations revealed a lag in the strengthening of limbic-cortical/subcortical connections in ADHD-model rats. This in turn related to impaired modularization of memory and reward-motivation associated regions. Using mathematical network analysis techniques such as single linkage hierarchical clustering and volume entropy, we observed left-lateralized connectivity in the ADHD-model rats at 6 weeks old. Our findings supported the maturational delay of metabolic connectivity in the SHR model of ADHD, and also suggested the possibility of impaired and compensative reconfiguration of information flow over the brain network.

## Introduction

Attention-deficit hyperactivity disorder (ADHD) is one of the most common brain developmental disorder characterized by typical symptoms of inattention, hyperactivity, and impulsivity. ADHD patients have not only problems with attention and executive function but also suffer from considerable memory impairment, especially for working memory and even long-term memory^1,2^. ADHD usually initiates during childhood or school-age^3^. Almost 35% of ADHD subjects become symptom-free within adolescence^4^. About the pathogenesis of ADHD, there has been controversy about whether ADHD is caused by permanent deviation from typical brain development or delayed normal maturation of the brain. The delayed maturation hypothesis was supported by structural evidence not only of longitudinal studies of the cortical thickening^5,6^, but also of a mega cross-sectional study of the volume of subcortical structures, including the amygdala, accumbens, and hippocampus^7^.

Meanwhile, ADHD subjects reveal not only these structural changes but also the dysfunction of interregional connectivity^8^. Pathophysiologic models of ADHD have suggested the dysfunction of the fronto-striatal circuit mediating executive functions or the insufficient suppression of the default mode network (DMN) during cognitively demanding situations^8,9^. However, regarding maturational changes of functional connectivity of ADHD, there is only a limited number of cross-sectional functional magnetic resonance imaging (fMRI) studies, which suggests developmental lag within DMN connectivity or in DMN connections with task-positive networks^10^. Considering the difficulty of repeated imaging in ADHD children, a preclinical longitudinal study in rats might help corroborate this ‘delayed maturation’ hypothesis or to discover more elaborate findings to be translated to explain the changes of brain networks in ADHD children.

In this investigation, we examined the developmental changes of whole-brain network longitudinally by acquiring brain F-18 fluorodeoxyglucose (FDG) positron-emission-tomography (PET) from ages of childhood (4 weeks old) to the period of ‘entry of puberty’ (6 weeks old) in a spontaneous hypertensive rat (SHR) model of ADHD and in its control that Wistar Kyoto rat (WKY). Because of the behavioral heterogeneity, we chose rats with ADHD-phenotype using three behavioral tests; marble burying test (MBT) and delay discounting task (DDT) as impulsivity tests and open field test (OFT) as a hyperactivity test. Burying of harmless objects has been considered compulsive-, anxiety-like, or impulsive behavior of rodents^11^. Even though it lacks illness specificity, MBT has been used as one of the impulsive burying indexes^12,13^. DDT was applied to compensate for the lack of specificity for impulsivity, which measures the tendency to prefer immediate rewards than larger but delayed rewards. Rats with impulsivity are intolerant to the forced waiting for a delayed reward^14^. OFT presents information about exploratory behavior in the new environment and often adopted as hyperactivity index in ADHD-model^15^.

One of most common approaches to assessing brain connectivity from brain imaging data is constructing a graph composed of nodes (i.e. voxels or brain regions) and edges representing connections among nodes. The connections are usually assessed based on statistical dependencies, (i.e. correlation) among neurophysiological signals of brain regions at a certain thresholding level^16^. The problem is that there is no general rule for the appropriate level of thresholding, and it affects to the results of graph parameters.

Topological data analysis is a relatively new mathematical framework based on the algebraic topology, which is useful to acquire intrinsic topological features^17^. Algebraic topology generally concerns the shape of topological spaces regardless of stretching and shrinking^18^. For example, the *k*-th Betti-number is topological information representing the number of k-dimensional holes in a topological space. The zeroth Betti-number (*β*_0_) represents the number of connected components in a given topological space. Creating a filtered graph from a weighted graph, which preserves the relational information of original weights and its ordering, is particularly useful to avoid arbitrariness of threshold^18^. The graph filtration was successfully applied to brain network analysis^19–21^.

The maturation status of brain connectivity over the age of the rat may be related to the integration of whole brain regions^22^. To evaluate the integration of brain networks, we used graph filtration^19^. During the graph filtration, we observed the change of the connected structure of a brain network by varying thresholds, which was related to hierarchical single linkage clustering. A barcode and persistence diagram are widely used in TDA. However, in this study, a *β*_0_-curve is used for quantifying and visualizing the change of connected structures in a brain network, because it has the same information as the barcode and persistence diagram of connected components in the graph filtration. We also quantified and visualized the local changes of brain network integration by single linkage matrix (SLM), and minimum spanning tree (MST).

The graph filtration showed the whole brain integration procedure; however, it did not show the network topology after all the brain regions were integrated. To solve this problem, we applied volume entropy and generalized Markov system (GMS) to a connected component that had all nodes in a network with the minimum number of edges, called the giant connected component (GCC) of the whole-brain metabolic network in this paper. The GMS with the volume entropy is a model for measuring information flow on a brain network. It found the volume entropy and edge capacity that measured network efficiency in the view of information flow and the amount and direction of information flow on edges, respectively^23^. Traditional approaches of directed brain networks have mainly considered effective connectivity where the direction of edges is estimated by causality between two nodes^16^. On the other hand, the direction in edge capacity was estimated from the local difference of topology in a network. In other words, while the traditional directed networks were mainly a method for network construction directly estimated from data, the proposed method applied to a network, not a data, in order to see the local difference of topology in a network.

**Figure 1:**
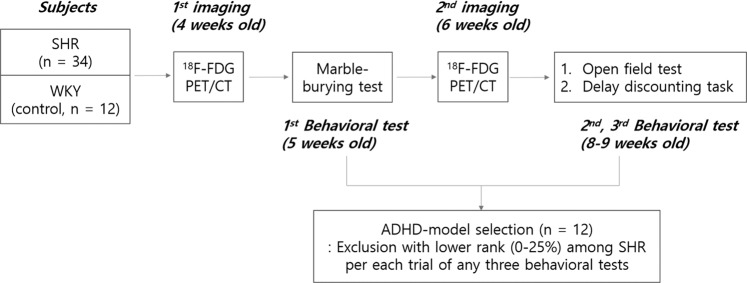
Scheme of rat experiments for FDG PET imaging and intervening behavioral tests. The overall scheme of the animal experiment was composed of two times of PET imaging at 4 weeks and 6 weeks of age and three behavioral tests, i.e., marble-burying test, open field test, and delay discounting task. Phenotypically positive ADHD-model rats were chosen later, with the behavioral tests blinded to the imaging results.

The details of the experimental design (Fig. 1), image preprocessing method and topological network analysis are in the method section.

## Results

### Behavioral characteristics of ADHD-model rats

**Figure 2:**
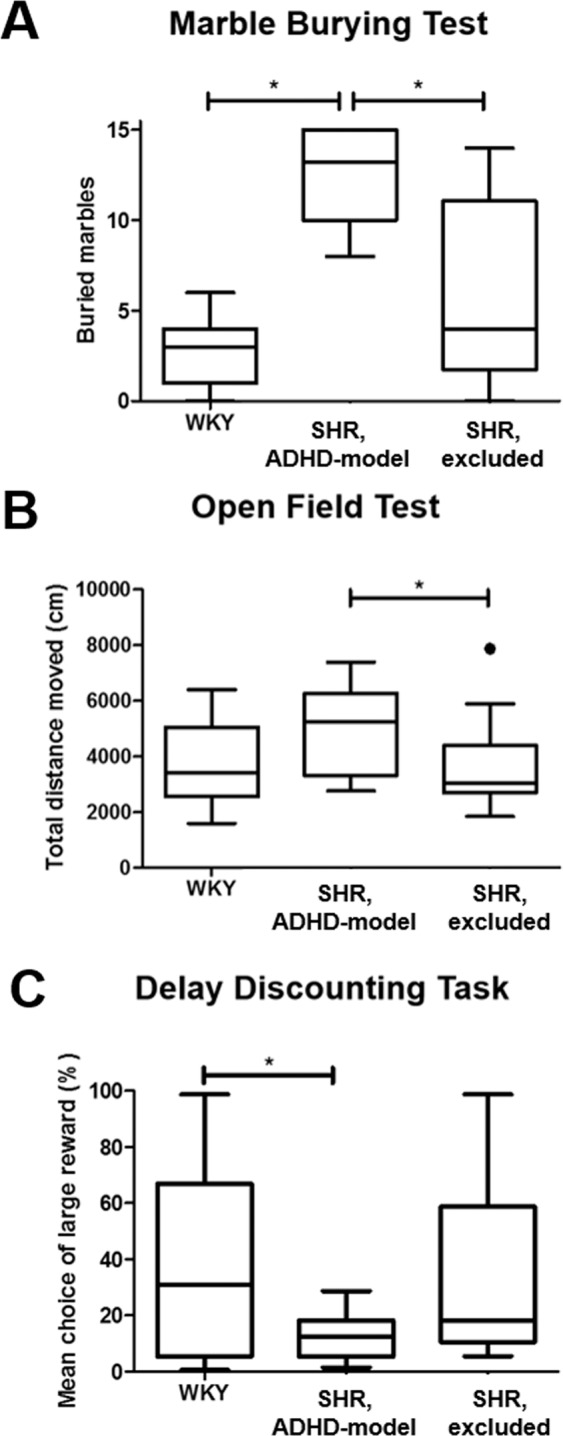
Behavioral tests representing ADHD-phenotype. ADHD-phenotype was tested by three behavioral tests: marble burying test (**A**), open field test (**B**), and delay discounting test (**C**).

The selected ADHD-model rats significantly buried more marbles than the control rats in the MBT at 5 weeks old (12.5 ± 2.6 *vs*. 2.8 ± 2.1; P < 0.001) (Fig. 2A). The ADHD-model rats moved more than the control rats in the OFT at 8–9 weeks old with a borderline significance (5.0 m ± 1.5 m *vs*. 3.6 m ± 1.3 m; P = 0.079) (Fig. 2B). In the DDT at 8–9 weeks old, the ADHD-model rats chose the immediate but small rewards more than the large but delayed rewards more frequently than the control rats (12.8% ± 8.2% *vs*. 35.8% ± 33.3%; P = 0.030) (Fig. 2C).

### Delayed maturation of limbic-cortical/subcortical connections

**Figure 3:**
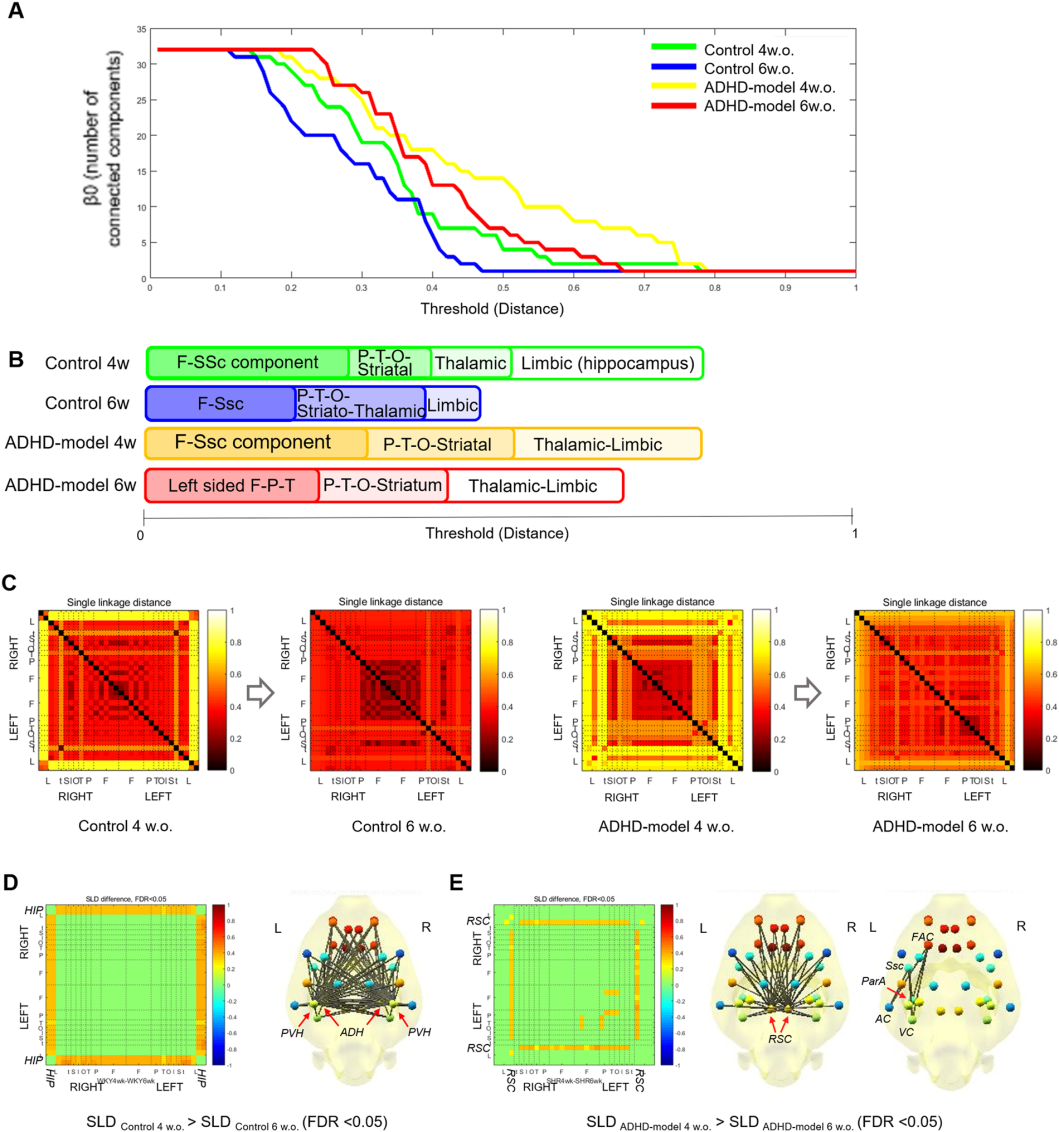
Brain network analysis with graph filtration during maturation. The changes in the number of connected components with threshold filtration were shown (**A**). The sequence of integration of a GCC during filtration was visualized in each group (**B**). Maturational changes of SLDs between region pairs were shown in the form of SLMs (**C**). The SLDs between hippocampus-cortical/subcortical pairs of regions were significantly getting closer in the control rats (WKY) during growth (FDR < 0.05) (**D**). The SLDs between RSC-cortical/subcortical pairs and between left-sided cortices of the ADHD-model rats (SHR_ADHD) were significantly getting closer during growth (FDR < 0.05) (**E**). Abbreviations: AC, auditory cortex; ADH, anterodorsal hippocampus; F, frontal; FAC, frontal association cortex; FDR, false discovery rate; GCC, giant connected component; HIP, hippocampus; O, occipital; P, parietal; ParA, parietal association cortex; PVH, posteroventral hippocampus; RSC, retrosplenial cortex; SLD, single linkage distance; SLM, single linkage matrix; Ssc, somatosensory cortex; T, Temporal; VC, visual cortex; w. or w.o., weeks old.

Curves of *β*_0_ which visualize the change in the number of connected components with filtration showed that GCC was made faster meaning stronger connections in the control rats than in the ADHD-model rats at the same ages. Also, GCC was made faster in older age than younger age in both groups of the control rats and ADHD-model rats (Fig. 3A). In detail, the control rats at 4 weeks old showed that the frontal and somatosensory cortices were strongly connected first during filtration. Subsequently, other temporo-parieto-occipital cortices, striatum, and limbic regions including the entorhinal cortex and retrosplenial cortex (RSC) were joined to the fronto-somatosensory component. The thalamus (THA) and hippocampus were the last nodes to construct the GCC. When the control rats grew up to 6 weeks old, the GCC was made faster and kept the consistency of the hierarchy of whole brain integration.

The integrating hierarchy of the GCC in the ADHD-model rats at 4 weeks old was similar to that of the control rats even though the integration of the temporo-parieto-occipital cortices, striatum, and limbic regions was slower. In the ADHD-model rats at 6 weeks old, the connections among the left-sided fronto-parieto-temporo-occipital cortices were stronger than those among the right-sided cortices. The limbic regions were integrated faster than in the ADHD-model rats of 4 weeks of age but still the last part integrated into the GCC (Fig. 3B,C). Dendrograms presenting a detailed connecting hierarchy were provided in Supplemental Fig. S1.

The cortical/subcortical connections with the hippocampus in the control rats were significantly stronger at 6 weeks old compared with at 4 weeks old (false discovery rate [FDR] < 0.05 by paired permutation) (Fig. 3D). Meanwhile, the ADHD-model rats at 6 weeks old had stronger connections among the left-sided cortices and between RSC and cortical/subcortical regions compared with at 4 weeks old (FDR < 0.05 by paired permutation) (Fig. 3E).

### Impaired modularization of memory and reward-motivation regions

**Figure 4:**
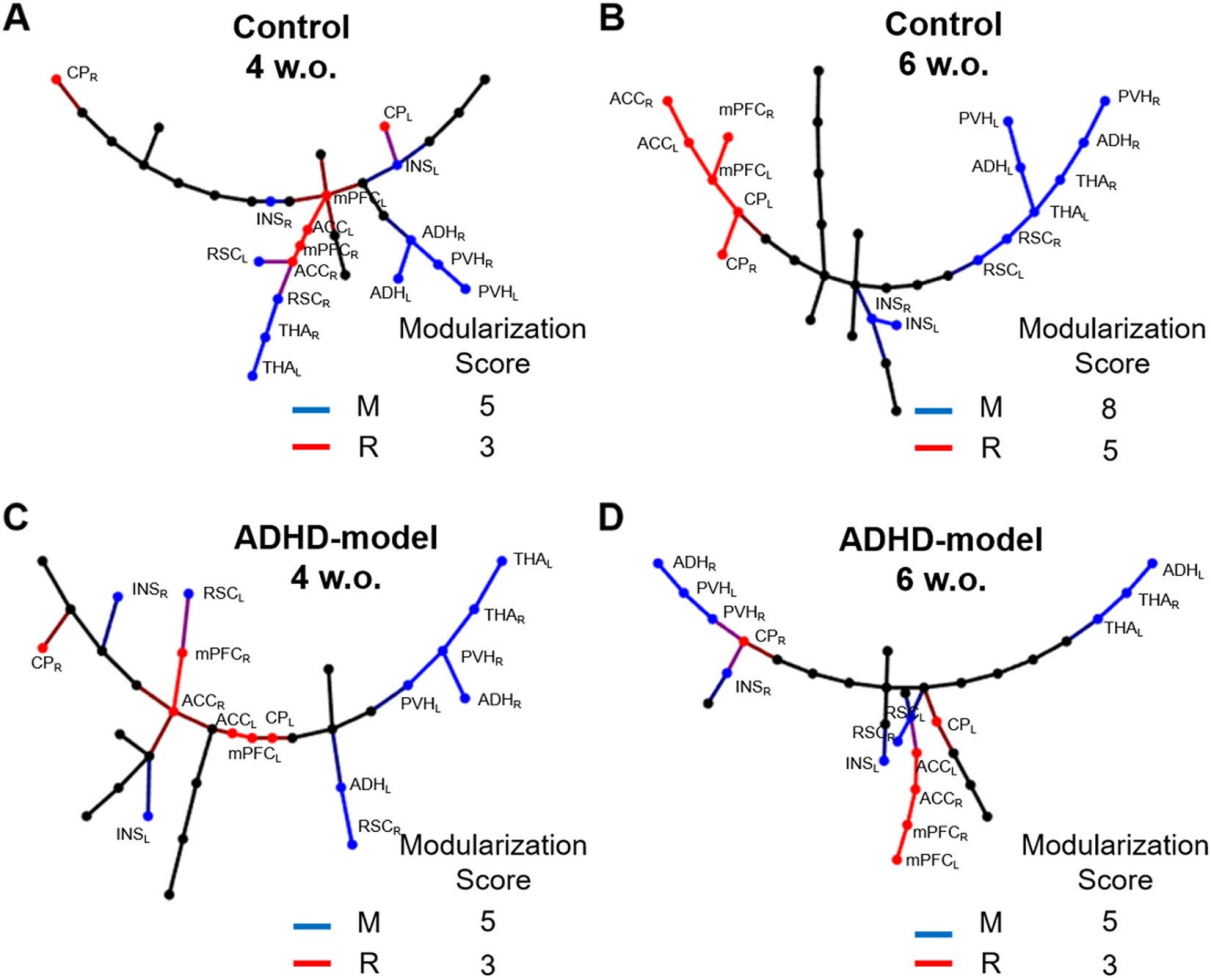
Modularization of memory and reward-motivation related regions on MSTs. The MSTs of control rats at the ages of 4 (**A**) and 6 weeks (**B**), and of ADHD-model rats at the ages of 4 (**C**) and 6 weeks (**D**) were visualized. The modularization score was counted as the number of directly connected edges between nodes belonging to the memory-related (blue) or the reward-motivation-related (red) regions. Abbreviations: ACC, anterior cingulate cortex; ADH, anterodorsal hippocampus; CP, caudoputamen; INS, insular cortex; L (small), left; mPFC, medial prefrontal cortex; MST, minimum spanning tree; M, memory-related regions; PVH, posteroventral hippocampus; R, reward-motivation-related regions; R (small), right; RSC (retrosplenial cortex); w.o., weeks old.

The memory-related nodes (bilateral anterodorsal hippocampus [ADH], posteroventral hippocampus [PVH], THA, RSC, and INS) and the reward-motivation-related nodes (bilateral ACCmedial prefrontal cortex [mPFC], caudoputamen [CP]) were modularized on the MSTs of control rats during growth. The number of directly connected edges between the memory-related nodes on the MSTs significantly increased in the control rats from 4 weeks to 6 weeks old (5 *vs*. 8, P < 0.05 by paired permutation) (Fig. 4A,B), but not in the ADHD-model rats (5 *vs*. 5, P > 0.10 by paired permutation) (Fig. 4C,D). The modularization trend was also observed in the reward-motivation regions consisting of CP, mPFC and ACC in the control rats with borderline significance (3 *vs*. 5, P = 0.10 by paired permutation) (Fig. 4A,B), not in the ADHD-model rats (3 *vs*. 3, P > 0.10 by paired permutation) (Fig. 4C,D).

### Global feature changes during development

**Figure 5:**
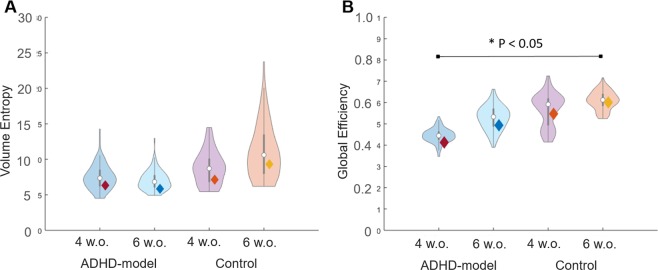
Volume entropy and global efficiency of brain graphs. The efficiency of brain graphs was assessed by volume entropy (**A**) and global efficiency (**B**). Rats with ADHD trait and younger age had less amount of volume entropy and less efficient brain graphs than control and older aged. Brain networks of both groups had been improved in regards to global efficiency, but only in control rats in regards to volume entropy. Note: Violin plots were results of 100-bootstrapping in each group. Abbreviation: w.o., weeks old.

There was an increasing trend of the volume entropy in the control rats during growth (P = 0.10 by paired permutation) but not in the ADHD-model rats (P > 0.10 by paired permutation) (Fig. 5A). In contrast, both groups showed the trend of increasing global efficiency during growth with a borderline significance (all P < 0.10 by paired permutation, respectively), and the ADHD-model rats’ brain networks had lower global efficiency than the control rats’ ones. There was a significant difference in global efficiency between the ADHD-model rats at 4 weeks old and control rats at 6 weeks old (P < 0.05 by 10,000 permutation) (Fig. 5B).

### Asymmetric information propagation

**Figure 6:**
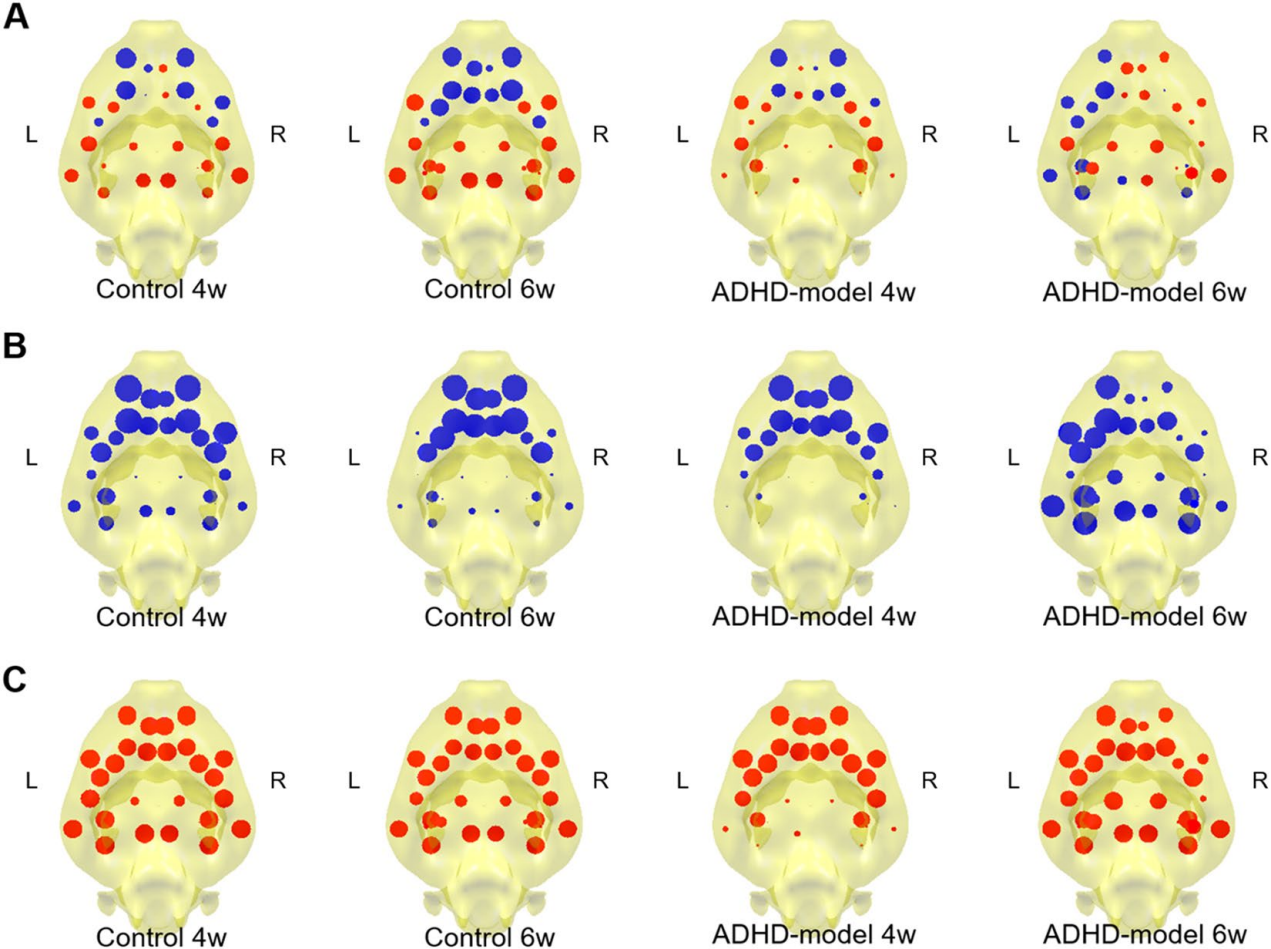
Mapping of stationary information flow at nodes. Node capacity maps of 4 groups that control rats of 4 and 6 weeks old and ADHD-model rats of 4 and 6 weeks old were visualized (**A**). Afferent node capacity (sum of afferent edge capacities to each node) (**B**) and efferent node capacity (sum of efferent edge capacities from each node) (**C**) were visualized as blue and red discs with variable size, respectivelyi. Thus, blue color in (**A**), means larger afferent node capacity than effent node capacity at a node and red color in (**A**) means larger efferent node capacity than afferent node capacity at a node. Abbreviations: L, left; R, right; w., weeks old.

Mapping node capacity on a directed graph of edge capacities representing information propagation showed a pattern that positive (larger sum of afferent edge capacities than sum of efferent edge capacities at a node) at anterior and negative (larger sum of efferent edge capacities than sum of afferent edge capacities at a node) at posterior in the control rats (Fig. 6A, ‘Control’). Now we will call ‘afferent node capacity’ meaning a sum of afferent edge capacities for a node (Fig. 6B) and ‘efferent node capacity’ meaning a sum of efferent edge capacities for a node (Fig. 6C). Node capacity is simply the difference between afferent and efferent node capacities (afferent node capacity minus efferent node capacity).

During development from 4 weeks to 6 weeks of age, there were little changes in node capacity map. In the ADHD-model rats at 4 weeks of age, the afferent and efferent node capacities in the posterior/subcortical nodes were all small, which meant the least participation of the nodes to the information propagation (Fig. 6A–C, ‘ADHD-model 4w.’). The ADHD-model rats at 6 weeks of age had a hemispherical asymmetric pattern of left-deviated information propagation of the brain network. When compared afferent and efferent node capacity maps with ADHD-model rats at 4 weeks of age, the ADHD-model rats at 6 weeks of age had less participation of left frontal areas and relatively more participation of posterior/subcortical areas **(**Fig. 6A–C, ‘ADHD-model 6w.’).

**Figure 7:**
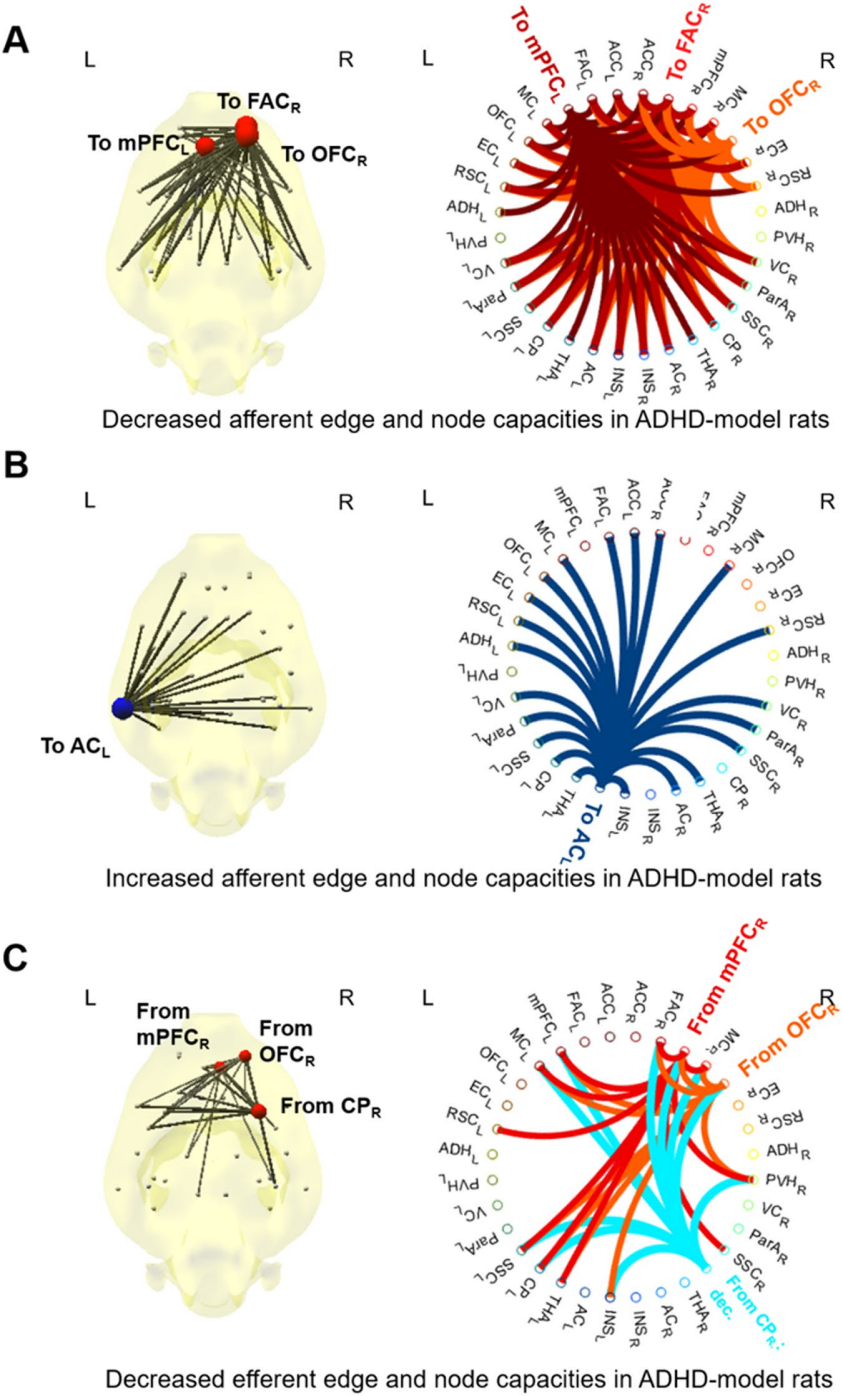
Nodes and edges with significantly different afferent or efferent capacities in the ADHD-model rats at 6 weeks old compared with the control rats at the same age. About afferent node capacity (sum of afferent edge capacities at a node), the ADHD-model rats’ was lower at right FAC, right OFC, and left mPFC (**A**), and higher at left AC significantly than the control rats at the same age (all FDR < 0.05) (**B**). About efferent node capacity (sum of efferent edge capacities at a node), the ADHD-model rats’ was lower at right mPFC, OFC and striatum (all FDR < 0.05) (**C**). Edges with significantly different edge capacities were also visualized (all FDR < 0.05) (**A**–**C**). Abbreviations: AC, auditory cortex; CP, caudoputamen; FDR, false discovery rate; mPFC, medial prefrontal cortex; OFC, orbitofrontal cortex. Note: See Supplemental Methods and Materials for details of regions of interest. The size and color (red/blue) of node disc on the brain diagram represented the amount of difference of afferent or efferent node capacity compared with the control rats. Each node had its color on circular plots, which indicated the destination of afferent edges in (**A,B**) or the starting points of efferent edges in (**C**).

The ADHD-model rats at 6 weeks old had significantly larger afferent node capacity at the left auditory cortex (AC) (FDR < 0.05 by 10,000 permutation) and significantly smaller afferent node capacity at the right frontal association cortex (FAC), right orbitofrontal cortex (OFC) and left mPFC compared with those of the control rats of the same age (FDR < 0.05 by 10,000 permutation) (Fig. 7A). The ADHD-model rats at 6 weeks old had significantly smaller efferent node capacities at right mPFC, OFC, and striatum than those of the control rats of 6 weeks age (FDR < 0.05 by 10,000 permutation) (Fig. 7B).

## Discussion

Our results supported the delayed maturation hypothesis by metabolic connectivity with topology analysis in the ADHD-rat model. Within the observation period until early puberty age, the ADHD-model rats had the maturation pattern of the metabolic network architecture in a delayed way, especially in the limbic and cortical/subcortical connections as well as the global efficiency. The modularization problems were found for the memory-related and reward-motivation-related regions in the ADHD-model rats, which might be associated with ADHD-behavior. Noteworthy, the ADHD-model rats at 6 weeks old had asymmetry in both of the brain connectivity architecture by the filtration framework and the information flow by the mathematical modeling. We interpret this asymmetry due to the abnormal developmental circuit(s) dysfunction/compensation in the ADHD-model rats.

From the integration hierarchy of whole-brain network, we could find the trend of the delayed maturation of the cortico-striato-thalamo-cortical (CSTC) circuits and large-scale networks including the parieto-temporo-occipital cortices. This trend of integration hierarchy of whole-brain network was once identified in typically developing children evaluated with the graph-filtration framework, which revealed faster wiring between the frontal and parietal cortices, followed by other cortices and subcortical structures^19^. The CSTC circuits have extensive connections with the parieto-temporo-occipital cortices, which compose large-scale brain networks outside CSTC circuits. Several parallel CSTC circuits, including the motor, associative, and limbic circuits have been known to be associated with ADHD^24^. Less mature CSTC circuits and large scale network observed in the ADHD-model rats matched well with previous fMRI human studies, which reported a decrease of resting-state connectivity of ADHD in the fronto-striatal, fronto-parietal, and temporo-parietal networks^25–28^.

The finding of weaker limbic-cortical/subcortical connections in the ADHD-model SHR rats was concordant with the result of a preclinical study in another ADHD model, Naples-High-Excitability (NHE) rats, on the resting-state functional MRI (rsfMRI). In this previous study, connectivity decreased between the hippocampus and entire cortex in the NHE rats compared with that in the Naples-Random-Bred controls^29^. A human fMRI study reported that the immature frontal-limbic activation pattern was the most characteristic pattern for discriminating the controls and ADHD children, which furthermore correlated with symptom severity^30^.

The asymmetry of information flow which appeared at 6-weeks of age in the ADHD-model rats was thought to be the result of both abnormality and its compensation process. The right frontal cortices and striatum were considered to be impaired regions in the ADHD-model because those regions had both decreased afferent and efferent node capacities, which means less contribution of those nodes to the information flow. However, the left hemisphere with the rich afferent information flow had a similar level of efferent information flow compared with other nodes, which indicates a compensative process, not an impaired process. We interpreted this as representing the asymmetry in treating information flow.

The finding as hemispherical asymmetry is consistent with the previous studies reporting abnormal brain laterality with the right hemisphere deficit in ADHD children^31–34^. ADHD often have right-sided spatial attentional deficits, which could have resulted from dysfunction within the right frontal-parietal network^35^. However, there have been contradictory results emphasizing the left-sided impairment in ADHD^36^. Our data support the right frontal deficit in ADHD.

The left-lateralized connectivity with the rich information flow on the left AC might be compensatory changes to the developmental impairment of the right frontal cortex. A human fMRI study reported greater activation of left hemispheric linguistic processing areas during forward digit span task in adults with ADHD^37^. Another human fMRI study has reported the association of higher temporal cortex activations and lower variability for post-error trial with Go/No-Go task in ADHD children, suggesting the compensatory recruitment of temporal area^38^. The compensatory process is observed in various developmental disorders, including autism^39^ or tic-disorders^40^ as well as ADHD^41^.

SHR is used popularly as a rat model of ADHD-combined type, as they develop typical behavioral characteristics of ADHD such as hyperactivity and inattention at 5 to 9 weeks of age^42^. SHR rats had smaller striatal volumes than control WKY rats^43^, which was a consistent finding in human ADHD^7^. Individuals of SHR show the variability of behavioral characteristics^44^. The behaviorally homogeneous group with inattention and hyperactivity was used as the ADHD-model rats in our study. Meanwhile, a resting-state fMRI study with SHR rats at 6 weeks old without assessment of behavioral characteristics has reported variations in the DMN under the different depth of isoflurane anesthesia^45^. In our study, the rats were anesthetized during the injection of FDG and were awake for waiting and anesthetized again during PET/CT imaging. FDG was taken up in the rats’ brains for 35–40 minutes while they were awake. Therefore, the issue of behavioral heterogeneity in SHR rats and the effect of anesthesia did not compromise the findings of our study.

Our preclinical study has several limitations, such as small sample size, which would have affected statistical power and thus higher false non-discovery. As the first study of this kind with 12 rats per each group, we did not attend to type II error and instead carefully performed statistical tests to control type I error using FDR-correction. Our study provides information about brain network maturation of ADHD-rat model till entry of puberty age; however ADHD in humans has quite prominent symptoms after puberty and early adulthood ages. Though connectivity analysis for these ages might be helpful to understand ADHD pathophysiology more, SHR spontaneously develops hypertension after they are adults (10–12 weeks of age), which is a confounding factor in ADHD-model^46^. Further studies with ADHD children are needed to validate our findings. It might or might not corroborate our findings in rats, either of which will unravel the similarity between the rat model and human ADHD or discordance.

In conclusion, our preclinical longitudinal study validated that delayed maturation underlies in the wiring of the metabolic network in the SHR model of ADHD during development from childhood to entry of adolescence. Asymmetry of information propagation over the ADHD brain network might be a composite result of impairment in the right fronto-striatal regions, and compensatory reconfiguration of brain network in left-sided strengthened. Topological approaches with graph filtration and mathematical modeling estimating information flow might be useful for unveiling connectivity problems in brain disorders.

## Methods

### Animal models and experimental design

All animal care and experiments for this research were approved by the Seoul National University Institutional Animal Care and Use Committee and the Kyung Hee University Institutional Animal Care and Use Committee. All experiments were performed in accordance with relevant guidelines and regulations regarding the care and the use of animals for the experimental procedures. Thirty-four SHRs and 12 WKYs were raised in a laboratory cage with a standard condition (22–24 °C, 12-hours light and dark cycle) with no restriction of standard feeding and water-drinking. All the rats underwent two times of brain FDG PET scans at 4 weeks old and 6 weeks old, which represent childhood and entry of puberty, respectively^47^. Behaviors, including hyperactivity and impulsivity, were checked with MBT at 5 weeks old, and OFT and DDT at 8 to 9 weeks old (Fig. 1). Rats belong to the lower quartile per each behavioral test in SHRs were excluded from the ADHD-model rats, which led to twelve ADHD-model rats matching the number of WKY rats. Whole-brain connectivity based on brain metabolic activity was analyzed for 4 groups as follows: (1) ADHD-model rats at 4 weeks old, (2) ADHD-model rats at 6 weeks old, (3) control rats at 4 weeks old, and (4) control rats at 6 weeks old.

### Behavioral tests

MBT was performed individually to reveal the rats’ degree of impulsivity. Rats were acclimated for 15 minutes before MBT. They were tested with 3 × 5 placed glass marbles for 15 minutes after the acclimatization. The number of buried marbles was counted after the removal of the rats from the cages. Burial of marble was determined when 50% or more of it was covered by bedding. OFT was performed individually to reveal rats’ hyperactivity by measuring the total distance of moving around for 30 minutes. The movement of rats was tracked by a video camera system installed above the open-field apparatus.

DDT measuring intolerance to delay was performed individually using previously described methods with minor changes^48,49^. Briefly, after habituation in the animal room without the restriction of feeding and sequential 2 days of food restriction, rats were trained for 5 days on two levers returning different amounts of food pellets per one press. A press on the right lever delivered a food pellet (about 45 mg) immediately (small and immediate reward), whereas a press on the left lever resulted in the delivery of five food pellets (large and delayed reward) later. After pellet delivery, the time-out period lasted 20 seconds, and the light was on during this period. During the testing phase for 4 days, a delay was sequentially increased for the large rewards over the test days (0, 10, 20, 30, and 40 seconds). Each test took 30 minutes. During adjustment of delay sessions, restricted feeding that 5 g of pellet per 100 g of body weight was allowed. The mean percentage of choice for the larger rewards with specific delays from 20 to 40 seconds was considered as the score of DDT.

### Brain PET scanning and reconstruction

Brain PET images were obtained using a dedicated small animal PET/computed tomography (CT) scanner (eXplore VISTA, GE Healthcare, WI) after overnight fasting. Before brain PET/CT scanning, rats were anesthetized by 2% isoflurane at 1.5–2 L/min oxygen flow for 5 minutes before the injection of FDG (150–220 MBq/kg) via a tail vein. Rats were awake and took a rest in a dark room till brain PET/CT scanning. A static brain PET scan was acquired for 20 minutes, 45 minutes after FDG injection. The energy window of PET scanning was 250–700 keV. PET images were reconstructed using the three-dimensional ordered-subsets expectation maximum algorithm with the correction of attenuation, random, and scatter. The voxel size of reconstructed PET images was 0.3875 × 0.3875 × 0.775 mm^3^.

### Preprocessing of brain FDG PET

Voxel size was rescaled by a factor of 10 in each dimension. The rescaled brain PET images were manually realigned to the Schiffer template of rat brain MRI T1 in PMOD2.7 (PMOD group, Zurich, Switzerland)^50^. Spatial alignment using non-linear registration on Statistical Parametric Mapping (SPM8, University College of London, London, UK) was applied with the Schiffer template of rat brain PET and binary brain mask. Global normalization of voxel counts was applied as the last step of preprocessing.

### Brain parcellation and distance matrix computation

Among the 58 predefined ROIs on the Schiffer template, 32 ROIs, including the cortices and subcortical structures, were selected as nodes to construct a brain metabolic network in each group. See the Supplemental Methods and Materials for the details of the 32 ROIs. The Pearson correlation coefficient (*r*_*ij*_, *r*_*ij*_ > 0) between two nodes (*p*_*i*_, *p*_*j*_) was computed to obtain a positive correlation matrix. A distance (*d*_*ij*_) between two nodes (*p*_*i*_, *p*_*j*_) was defined as the following:

$${d}_{ij}=\sqrt{1-{r}_{ij}}$$

### Graph filtration

We used a multiscale approach to analyze networks to avoid fixing the threshold of distance^18^. In this study, we performed graph filtration, which decomposed a weighted network into unweighted networks at many possible thresholds. We found the connected components and the number of connected components, denoted by *β*_0_ of each unweighted network. The *β*_0_ decreased from the number of nodes in a network to one by merging two connected components into a connected component by increasing the threshold. When *β*_0_ = 1 at the minimum threshold, we called the connected component a GCC here. The change of *β*_0_ with respect to threshold is visualized by *β*_0_-curve. The *β*_0_-curve had the same information as the barcode of connected components. All bars in the barcode of connected components always start from zero in a network, and only the end of bars which corresponds to the death of connected components has the information of the change of connected structures. The *β*_0_-curve is obtained by connecting the end of bars in the barcode.

The threshold of distance when two connected components were merged into a connected component during the graph filtration is called a single linkage distance (SLD) between the two connected components. If the two connected components are denoted by *A* and *B* ($A\cap B=\varnothing$), then, the SLD between *A* and *B* is defined by

$$d(A,B)={{\rm{\min }}}_{x\in A,y\in B}d(x,y)\,and\,d(A,B)\ge d({C}_{1},{C}_{2})\,for\,any\,{C}_{1},{C}_{2}\subseteq A\,or\,B.$$

The SLD between two nodes in A and B is defined by^51^

$$d(x,y)=d(A,B)\,for\,all\,x\in A,y\in B.$$

When the number of nodes in a network is *p*, SLM is a *p*-by-*p* matrix of which element is an SLD. The *β*_0_ was counted along the filtration to make a dendrogram, which is equivalent to an SLM. An example of counting *β*_0_ and constructing an SLM is provided in Supplemental Fig. S2.

### Minimum spanning tree

Minimum spanning tree (MST) is a subset of a weighted network that has all nodes and the subset of edges which only connect $\hat{x}$ and $\hat{y}$ that satisfies

$$(\hat{x},\hat{y})={\rm{\arg }}\,{{\rm{\min }}}_{x\in A,y\in B}d(x,y)\,and\,d(\hat{x},\hat{y})=d(A,B).$$

The MST of a weighted network is a network that has the minimum number of edges that have the same SLM of the weighted network^51^. Therefore, it is easier to see the modular structure of a weighted network.

We evaluated modularization of the reward-motivation system (CP, mPFC, and ACC) and memory system (anterodorsal hippocampus [ADH], posteroventral hippocampus [PVH], RSC, THA, and insula [INS]) during growth by counting the number of a direct connection between nodes included in each system^52^.

### Volume entropy, and edge and node capacities

The graph filtration shows the change of the connected structure of a weighted network until constructing a GCC. To quantify the pattern of information flow of the weighted network constructing a GCC, we used the GMS of volume entropy.

The GMS is the generalization of the Markov chain defined on edges. In our GMS, an edge from *v* to *w* is different from an edge from *w* to *v* for any two nodes *v* and *w* in a network. Therefore, when the number of nodes is *p*, the number of all possible edges is *q* = *p*(*p* − 1). Moreover, the GMS assumes that the sum of edge weights in a weighted network is equal to 2. Then, the edge-transition matrix is defined by

$$L(h)=[{L}_{ef}={a}_{ef}{e}^{-hl(f)}]\in {R}^{q\times q},$$

where *a*_*ef*_ is 1 if an edge *e* is connected to an edge *f* in the network, 0, otherwise, *h* is a nonnegative constant, and *l(f)* is the weight of the edge *f*^23,53^.

Unlike random walk in Markov chain, the state transition by *L(h)* is from *i* + 1 to *I* such that

$${z}_{i}=L(h){z}_{i+1},$$

where *z*_*i*_ shows the state of *q* edges at the *i*th step. When $z={z}_{i}={z}_{i+1}$, the GMS is stationary, and *z*, the eigenvector of *L*(*h*) corresponding to an eigen value of 1, is called the stationary state of *q* edges, and *h* satisfying $z=L(h)z$ is called volume entropy.

The *z* can be represented in a matrix form such that $Z=[{z}_{vw}]$, where the (*v*, *w*)th element is the stationary state of an edge from a node *v* to a node *w* in *z*. The *Z* is called an edge capacity matrix. The afferent node capacity of a node *w* is obtained by the sum of the column vectors of *Z*, i.e., $\sum _{v}{z}_{vw}$, and the efferent node capacity of a node *v* is obtained by the sum of the row vectors of *Z*, i.e., $\sum _{w}{z}_{vw}$. The node capacity of a node *v* is obtained by the difference between the afferent and efferent node capacities, i.e., $\sum _{v}{z}_{wv}-\sum _{v}{z}_{vw}$ ^23^.

### Statistics

To examine the statistical differences of the graph parameters, including SLD, modulation score, volume entropy, global efficiency, edge capacity, and afferent and efferent node capacities, we applied the permutation test, which is a subset of non-parametric tests. In the unpaired permutation tests between the ADHD-model and control groups, individual PET images were randomly shuffled from a single mixed data set to construct two pseudo-groups which had the same number of subjects to the original data sets (*n* = 12) per each iteration. The graph parameters were calculated for the resampled pseudogroups in each iteration. The permuted distributions of the differences of the graph parameters between the pseudogroups were obtained via 10,000 times of iteration of the process, which were used to test the significance of the differences of the graph parameters between original groups with a two-tail P-value < 0.05 (Supplemental Fig. S3). In the paired permutation tests between 4 weeks old and 6 weeks old of the ADHD-model or control group, 12 times of random shuffling were available between paired data. Therefore, the paired permutation test with the enumeration of all the possible distinct 12-paired permutations (2^12^ = 4,096 times) was done to test developmental changes of graph parameters of each group from 4 weeks old to 6 weeks old^54^. Multiple comparison problem was controlled as an FDR less than 0.05 using fdr_bh function with the Benjamini-Hochberg procedure in MATLAB^55^.

## Supplementary information

Supplemental Figures.

Supplemental Methods and Materials.
