# Supplementary material for: Maturational delay and asymmetric information flow of brain connectivity in SHR model of ADHD revealed by topological analysis of metabolic networks
Source: Sci Rep. 2020 Feb 21;10:3197. doi: 10.1038/s41598-020-59921-4 (PMC7035354; doi:10.1038/s41598-020-59921-4)
Supplement: Supplementary file 1 — Supplemental Figures. [file 41598_2020_59921_MOESM1_ESM.docx]

**Maturational delay and asymmetric information flow of brain connectivity in SHR model of ADHD revealed by topological analysis of metabolic networks**

Seunggyun Ha^1,2^, Hyekyoung Lee^1,3^, Yoori Choi^1^, Hyejin Kang^1,4^, Se Jin Jeon^5^, Jong Hoon Ryu^5,6^, Hee Jin Kim^7^, Jae Hoon Cheong^7^, Seonhee Lim^8^, Bung-Nyun Kim^9*^, Dong Soo Lee^1,10*^

^1^Department of Nuclear Medicine, Seoul National University College of Medicine, Seoul, Republic of Korea;

^2^Division of Nuclear Medicine, Department of Radiology, Seoul ST. Mary’s Hospital, The Catholic University of Korea, Seoul, Republic of Korea;

^3^Biomedical Research Institute, Seoul National University Hospital, Seoul, Republic of Korea;

^4^BK21 Plus Global Translational Research on Molecular Medicine and Biopharmaceutical Sciences, Seoul National University, Seoul, Republic of Korea;

^5^Department of Oriental Pharmaceutical Science, College of Pharmacy, Kyung Hee University, Seoul, Republic of Korea;

^6^Department of Life and Nanopharmaceutical Science, College of Pharmacy, Kyung Hee University, Seoul, Republic of Korea,

^7^Department of Pharmacy, Uimyung Research Institute for Neuroscience, Sahmyook University, Seoul, Republic of Korea;

^8^Department of Mathematical Sciences, Seoul National University, Seoul, Republic of Korea;

^9^Division of Child and Adolescent Psychiatry, Department of Psychiatry, Seoul National University College of Medicine, Seoul, Republic of Korea;

^10^Department of Molecular Medicine and Biopharmaceutical Sciences, Graduate School of Convergence Science and Technology, and College of Medicine or College of Pharmacy, Seoul National University, Seoul, Republic of Korea

**[Correspondence and Reprint Request]**

^*^To whom correspondence should be addressed

Dong Soo Lee, M.D., Ph.D.

Department of Nuclear Medicine, Seoul National University College of Medicine

28 Yongon-Dong, Jongno-Gu, Seoul, 110-744, Korea

E-mail: [dsl@plaza.snu.ac.kr](mailto:dsl@plaza.snu.ac.kr)

Bung-Nyun Kim, M.D., Ph.D.

Department of Psychiatry and Behavioral Science, Seoul National University College of Medicine

28 Yongon-Dong, Jongno-Gu, Seoul, 110-744, Korea

E-mail: [kbn1@snu.ac.kr](mailto:kbn1@snu.ac.kr)

***Short/running:*** Brain network topology analysis in ADHD rat model

***Keywords:*** ADHD, delayed maturation hypothesis, metabolic connectivity, graph filtration, directed graph, volume entropy

***Supplemental Information***

**SUPPLEMENTAL FIGURES**

**
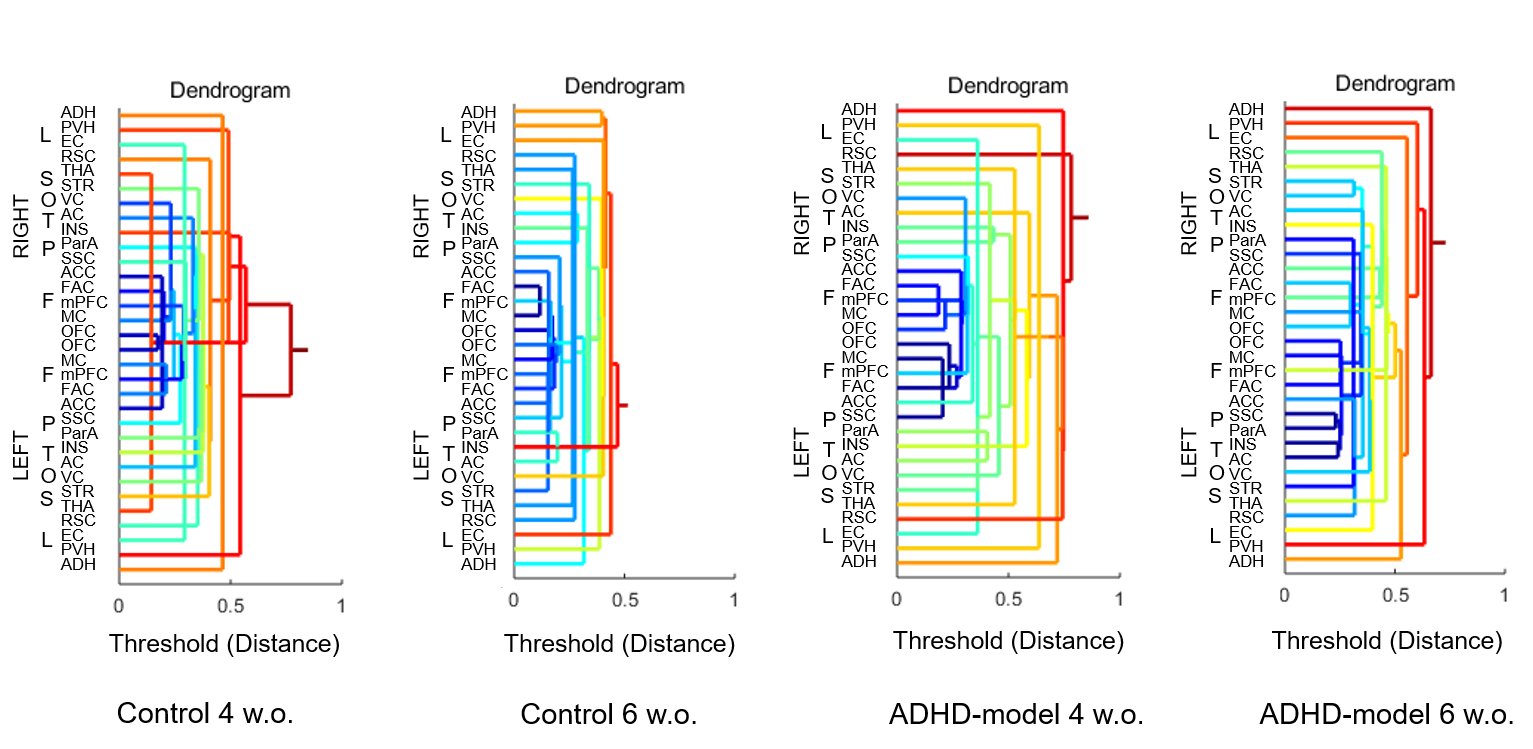
**

**Figure S1. Dendrogram visualizing sequential wiring of ROIs in filtration**

Dendrogram visualized sequential wiring during threshold filtration in each group. Frontal and somatosensory cortices were wired early and followed by other cortices and striatum in general. Thalamic and limbic regions were wired at last. During maturation, this trend was kept but wired faster. Meanwhile, ADHD-model at 6 weeks old had left-lateralized cortical wiring.


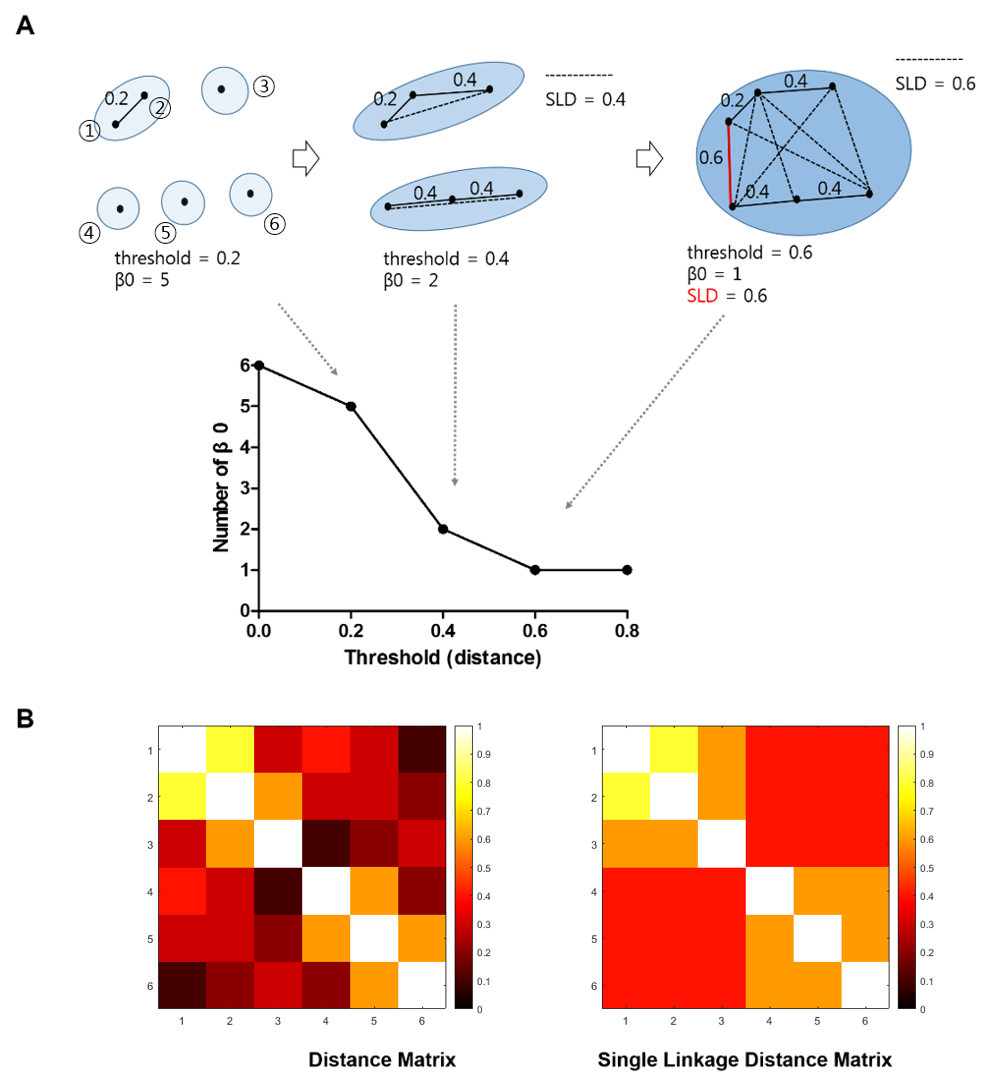


**Figure S2. Illustration of computing single linkage distance matrix via persistent homology**

This figure explains the concept of persistent homology by using an example of a weighted graph with 6 nodes. Persistent homology obtains the number of connected components, Betti-0 (β0), which quantifies 0-dimensional topological invariant of network. With the distance threshold of 0, the number of β0 is same to the number of regions of interest (ROIs). While changing thresholds for connection of nodes from strict to loose, β0 has values from the number of ROIs to 1. The changes in β0 are visualized by barcodes and dendrograms. While changing thresholds, a graph of monotonically decreasing β0 from the number of ROIs to one is referred as a barcode (A). The dendrogram shows the hierarchical connection between ROIs along the changing thresholds, which is the version before shuffling to make the barcode. The filtration value which allows a connection between two brain regions (separately connected components) is defined as single linkage distance (SLD) of newly connected nodes. The SLDs between all nodes can be displayed in the single linkage distance matrix (SLM) (B). Barcodes, single linkage dendrograms, SLMs, and minimum spanning tree are all the same unified subgraph calculated to yield the overall shortest edges by choosing the most important edges between nodes during graph filtration.


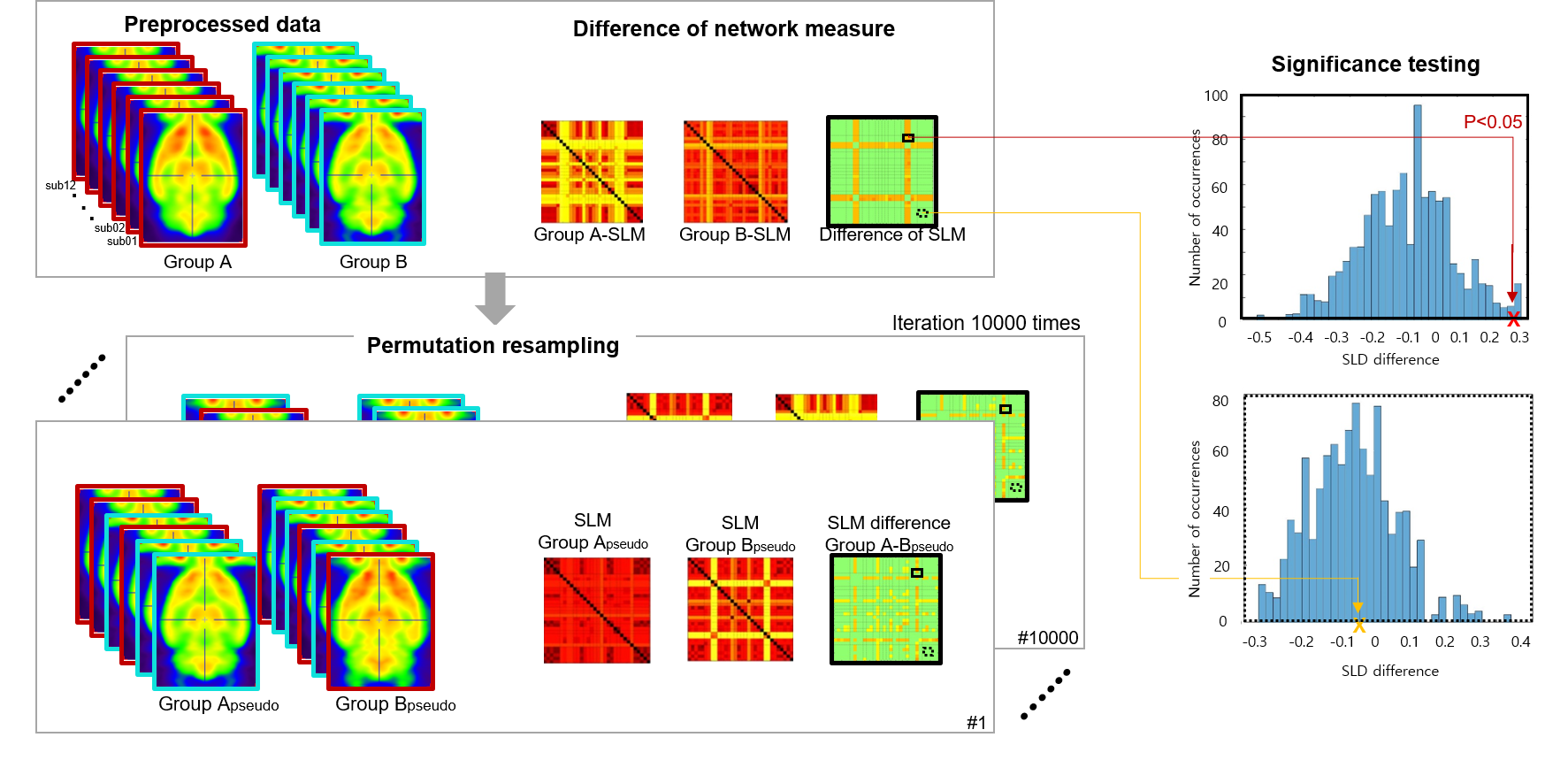


**Figure S3. Demographic illustration of permutation tests for comparison of metabolic connectivity.**

This figure illustrates the statistical comparison via 10,000-times permutation for the difference of single linkage distance matrix from two different groups, A and B. Single linkage distance matrices were generated from 10,000 randomly permuted groups, and the difference matrices of single linkage distance were obtained in each permutation. Type I errors were determined by the comparison of the difference of single linkage distance from observed data with the distribution of that of randomly permuted data.
