# Supplementary material for: Maturational delay and asymmetric information flow of brain connectivity in SHR model of ADHD revealed by topological analysis of metabolic networks
Source: Sci Rep. 2020 Feb 21;10:3197. doi: 10.1038/s41598-020-59921-4 (PMC7035354; doi:10.1038/s41598-020-59921-4)
Supplement: Supplementary file 2 — Supplemental Methods and Materials. [file 41598_2020_59921_MOESM2_ESM.docx]

**Maturational delay and asymmetric information flow of brain connectivity in SHR model of ADHD revealed by topological analysis of metabolic networks**

Seunggyun Ha^1,2^, Hyekyoung Lee^1,3^, Yoori Choi^1^, Hyejin Kang^1,4^, Se Jin Jeon^5^, Jong Hoon Ryu^5,6^, Hee Jin Kim^7^, Jae Hoon Cheong^7^, Seonhee Lim^8^, Bung-Nyun Kim^9*^, Dong Soo Lee^1,10*^

^1^Department of Nuclear Medicine, Seoul National University College of Medicine, Seoul, Republic of Korea;

^2^Division of Nuclear Medicine, Department of Radiology, Seoul ST. Mary’s Hospital, The Catholic University of Korea, Seoul, Republic of Korea;

^3^Biomedical Research Institute, Seoul National University Hospital, Seoul, Republic of Korea;

^4^BK21 Plus Global Translational Research on Molecular Medicine and Biopharmaceutical Sciences, Seoul National University, Seoul, Republic of Korea;

^5^Department of Oriental Pharmaceutical Science, College of Pharmacy, Kyung Hee University, Seoul, Republic of Korea;

^6^Department of Life and Nanopharmaceutical Science, College of Pharmacy, Kyung Hee University, Seoul, Republic of Korea,

^7^Department of Pharmacy, Uimyung Research Institute for Neuroscience, Sahmyook University, Seoul, Republic of Korea;

^8^Department of Mathematical Sciences, Seoul National University, Seoul, Republic of Korea;

^9^Division of Child and Adolescent Psychiatry, Department of Psychiatry, Seoul National University College of Medicine, Seoul, Republic of Korea;

^10^Department of Molecular Medicine and Biopharmaceutical Sciences, Graduate School of Convergence Science and Technology, and College of Medicine or College of Pharmacy, Seoul National University, Seoul, Republic of Korea

**[Correspondence and Reprint Request]**

^*^To whom correspondence should be addressed

Dong Soo Lee, M.D., Ph.D.

Department of Nuclear Medicine, Seoul National University College of Medicine

28 Yongon-Dong, Jongno-Gu, Seoul, 110-744, Korea

E-mail: [dsl@plaza.snu.ac.kr](mailto:dsl@plaza.snu.ac.kr)

Bung-Nyun Kim, M.D., Ph.D.

Department of Psychiatry and Behavioral Science, Seoul National University College of Medicine

28 Yongon-Dong, Jongno-Gu, Seoul, 110-744, Korea

E-mail: [kbn1@snu.ac.kr](mailto:kbn1@snu.ac.kr)

***Short/running:*** Brain network topology analysis in ADHD rat model

***Keywords:*** ADHD, delayed maturation hypothesis, metabolic connectivity, graph filtration, directed graph, volume entropy

***Supplemental Information***

**SUPPLEMENTAL METHODS AND MATERIALS**

**Analyzed Regions-of-Interest on the Rat Template (Schiffer)**

| No. | Full name | Short name | Location (Abbreviation) | RIGHT/LEFT |
| --- | --- | --- | --- | --- |
| 1 | Anterodorsal Hippocampus, Right | ADH_R_ | Limbic (L) | RIGHT |
| 2 | Posteroventral Hippocampus, Right | PVH_R_ | Limbic (L) | RIGHT |
| 3 | Entorhinal Cortex, Right | EC_R_ | Limbic (L) | RIGHT |
| 4 | Retrosplenial Cortex, Right | RSC_R_ | Limbic (L) | RIGHT |
| 5 | Thalamus Whole, Right | THA_R_ | Thalamic (t) | RIGHT |
| 6 | Caudate Putamen, Right | CP_R_ | Striatal (S) | RIGHT |
| 7 | Insular Cortex, Right | INS_R_ | Insula (I) | RIGHT |
| 8 | Visual Cortex, Right | VC_R_ | Occipital (O) | RIGHT |
| 9 | Auditory Cortex, Right | AC_R_ | Temporal (T) | RIGHT |
| 10 | Parietal Association Cortex, Right | ParA_R_ | Parietal (P) | RIGHT |
| 11 | Somatosensory Cortex, Right | SSC_R_ | Parietal (P) | RIGHT |
| 12 | Anterior Cingulate Cortex, Right | ACC_R_ | Frontal (F) | RIGHT |
| 13 | Frontal Association Cortex, Right | FAC_R_ | Frontal (F) | RIGHT |
| 14 | Medial Prefrontal Cortex, Right | mPFC_R_ | Frontal (F) | RIGHT |
| 15 | Motor Cortex, Right | MC_R_ | Frontal (F) | RIGHT |
| 16 | Orbitofrontal Cortex, Right | OFC_R_ | Frontal (F) | RIGHT |
| 17 | Orbitofrontal Cortex, Left | OFC_L_ | Frontal (F) | LEFT |
| 18 | Motor Cortex, Left | MC_L_ | Frontal (F) | LEFT |
| 19 | Medial Prefrontal Cortex, Left | mPFC_L_ | Frontal (F) | LEFT |
| 20 | Frontal Association Cortex, Left | FAC_L_ | Frontal (F) | LEFT |
| 21 | Anterior Cingulate Cortex, Left | ACC_L_ | Frontal (F) | LEFT |
| 22 | Somatosensory Cortex, Left | SSC_L_ | Parietal (P) | LEFT |
| 23 | Parietal Association Cortex, Left | ParA_L_ | Parietal (P) | LEFT |
| 24 | Auditory Cortex, Left | AC_L_ | Temporal (T) | LEFT |
| 25 | Visual Cortex, Left | VC_L_ | Occipital (O) | LEFT |
| 26 | Insular Cortex, Left | INS_L_ | Insula (I) | LEFT |
| 27 | Caudate Putamen, Left | CP_L_ | Striatal (S) | LEFT |
| 28 | Thalamus Whole, Left | THA_L_ | Thalamic (t) | LEFT |
| 29 | Retrosplenial Cortex, Left | RSC_L_ | Limbic (L) | LEFT |
| 30 | Entorhinal Cortex, Left | EC_L_ | Limbic (L) | LEFT |
| 31 | Posteroventral Hippocampus, Left | PVH_L_ | Limbic (L) | LEFT |
| 32 | Anterodorsal Hippocampus, Left | ADH_L_ | Limbic (L) | LEFT |
